# Silk-coated PLGA non-spherical microparticles for simvastatin delivery in rat maxillofacial bone regeneration

**DOI:** 10.1093/rb/rbag053

**Published:** 2026-03-10

**Authors:** Junjiang Zhang, Yuqi Chang, Yiwen Zhou, Yang Zhou, Jiayin Li, Feng Wang, Zhimin Zhou, Lei Sui, Yanjing Li

**Affiliations:** Tianjin Medical University School and Hospital of Stomatology, Tianjin 300070, China; Tianjin Key Laboratory of Oral Soft and Hard Tissues Restoration and Regeneration, Tianjin 300070, China; Tianjin Medical University School and Hospital of Stomatology, Tianjin 300070, China; Tianjin Key Laboratory of Oral Soft and Hard Tissues Restoration and Regeneration, Tianjin 300070, China; Tianjin Medical University School and Hospital of Stomatology, Tianjin 300070, China; Tianjin Key Laboratory of Oral Soft and Hard Tissues Restoration and Regeneration, Tianjin 300070, China; Tianjin Medical University School and Hospital of Stomatology, Tianjin 300070, China; Tianjin Key Laboratory of Oral Soft and Hard Tissues Restoration and Regeneration, Tianjin 300070, China; Tianjin Medical University School and Hospital of Stomatology, Tianjin 300070, China; Tianjin Key Laboratory of Oral Soft and Hard Tissues Restoration and Regeneration, Tianjin 300070, China; Tianjin Key Laboratory of Oral Soft and Hard Tissues Restoration and Regeneration, Tianjin 300070, China; Department of Genetics, School of Basic Medical Sciences, Tianjin 300070, China; Biomedical Barriers Research Center, Institute of Biomedical Engineering, Chinese Academy of Medical Sciences & Peking Union Medical College, Tianjin 300192, China; Tianjin Medical University School and Hospital of Stomatology, Tianjin 300070, China; Tianjin Key Laboratory of Oral Soft and Hard Tissues Restoration and Regeneration, Tianjin 300070, China; Tianjin Medical University School and Hospital of Stomatology, Tianjin 300070, China; Tianjin Key Laboratory of Oral Soft and Hard Tissues Restoration and Regeneration, Tianjin 300070, China

**Keywords:** PLGA non-spherical particles, morphology, simvastatin, local delivery system, maxillofacial bone regeneration

## Abstract

Maxillofacial bone defects present a major challenge in clinical practice. Bone grafting and bone tissue engineering are limited by various side effects and potential risks, while local drug delivery is regarded as a convenient and efficient strategy for bone repair and regeneration. However, for bone tissue regeneration, a drug delivery system should not only release osteogenic drugs but also possess an appropriate morphology to provide structural support for cell proliferation and differentiation. Polymeric non-spherical counterparts may offer new osteogenic properties through altered cellular interactions, biodistribution, and immune responses, compared with spherical drug carriers. Poly(lactic-co-glycolic acid) (PLGA) has been widely used to prepare various drug carriers due to its excellent biocompatibility and controllable biodegradation. Shape regulation, surface modification and drug incorporation are effective approaches to impart distinct functions to PLGA particles for various biomedical applications. However, PLGA has inherent limitations, including poor hydrophilicity, acidic degradation products and a lack of functional ligands for cell attachment, which hinder its effectiveness in bone regeneration. To solve these problems, natural proteins were utilized to improve the bone/cartilage regeneration of polymeric particles/scaffolds through surface modification or physical complexation due to their excellent biocompatibility and biodegradability. In this study, we successfully developed simvastatin (SIM)-loaded disc-shaped PLGA microparticles with coarse surface (dPLGA) using W_1_/O/W_2_ followed by silk fibroin (SF) coating for hydrophilicity and biocompatibility improvement. *In vitro* and *in vivo* experiments revealed that SF-modified SIM-loaded dPLGA (SF-SIM@dPLGA) significantly promoted the osteogenic differentiation of stem cells and bone regeneration in cranial bone defects, indicating the superior osteoconductive and osteoinductive advantages arising from the physical morphology of particles and chemical effects. Additionally, SF-SIM@dPLGA demonstrated improved tissue adhesion and hemostatic ability. This novel PLGA non-spherical particle-based local delivery system provides a feasible strategy for practical maxillofacial bone repair due to the simple preparation procedures and enhanced bone regeneration performance.

## Graphical abstract

**Graphical Abstract:**
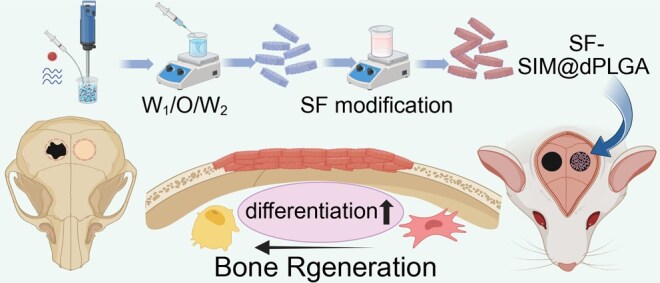

## Introduction

Maxillofacial bone defects caused by trauma, tumors or infection pose a substantial challenge in clinical practice. Although autogenous bone is considered the gold standard for bone defect treatment, both low availability and the corresponding donor site morbidity become inevitable limitations in clinical applications [1, 2]. The use of allogeneic bone is also constrained by risks of immune rejection, disease transmission and psychological distress in patients [3]. To address these limitations of bone grafts, tissue engineering strategies or local drug delivery systems have been developed to repair various bone defects. For tissue engineering, cell culture and differentiation as well as additional surgeries have to be considered for practical bone repair. Compared to cell-based bone regeneration, local drug delivery is an alternative method that can contribute to bone repair [4, 5]. Especially, recruitment of endogenous stem cells appears to have better advantages in the process of bone regeneration in comparison with exogenous stem cell utilization due to the high cost and potential risk of tumorigenesis [6]. Therefore, it is necessary to design a particle-based platform for drug delivery and stem cell recruitment *in vivo* simultaneously for maxillofacial bone regeneration.

Poly(lactic-co-glycolic) acid (PLGA) scaffolds or vehicles have been recognized as a potential candidate material for bone regeneration due to their exceptional biocompatibility, modifiability, degradability and efficient drug-loading capacity [7, 8]. PLGA spheres have been commonly studied as drug carriers and have shown a significant effect in bone regeneration by enhancing the bioavailability, stability and half-life of bone-promoting medications [9–11]. Non-spherical counterparts can offer new properties through altered cellular interactions, biodistribution and immune response, compared to traditional spherical drug carriers [12]. Non-spherical particles with specific curvature and aspect ratios could reduce phagocytosis by immune cells and alter their distribution on organ surfaces, including blood vessels, lungs and digestive tracts [13, 14]. Besides, PLGA disc-shaped microparticles enable sustained drug release while reducing potential side effects, thereby prolonging drug-retention time and improving *in vivo* pharmacokinetic study results [15]. The release profile of simvastatin (SIM), a typical cholesterol-lowering and osteoinductive drug, from PLGA non-spherical particles was faster *in vitro* than that of spherical counterparts, obviously due to stronger swelling of disc-like particles [16]. Moreover, wrinkled non-spherical particles were in favor of cell attachment for potential biomedical applications, and microscale pores of scaffolds or vehicles can induce the maturation and remodeling of new bone [17, 18]. Additionally, disc-shaped particles with a large size have lower curvature, which can provide a more stable platform, potentially promoting endogenous cell spreading and growth [19]. Therefore, PLGA non-spherical particles are a promising technology to offer new properties in maxillofacial bone regeneration. Nevertheless, there is no doubt that PLGA has inherent limitations, including poor hydrophilicity, acidic degradation products and a lack of functional ligands for cell attachment, which hinder its effectiveness in bone regeneration. To solve these problems, silk fibroin (SF), a commonly used natural protein, was utilized to improve the bone/cartilage regeneration of polymeric particles/scaffolds through surface modification or physical complex due to its excellent biocompatibility and biodegradability [20–25]. SF can enhance the water-absorption capacity of PLGA and effectively mitigate the adverse effects caused by the release of acidic degradation by-products [26]. Besides, SF coating enhanced specific sites for hydroxyapatite deposition on PLGA porous microspheres, promoted stem cell adhesion on porous surfaces and accelerated periodontal regeneration [27, 28]. Therefore, it is necessary to investigate SF-coated PLGA non-spherical particles for bone regeneration by means of integration of both the physical morphology of particles and the osteogenic drugs incorporation.

**Figure 1:**
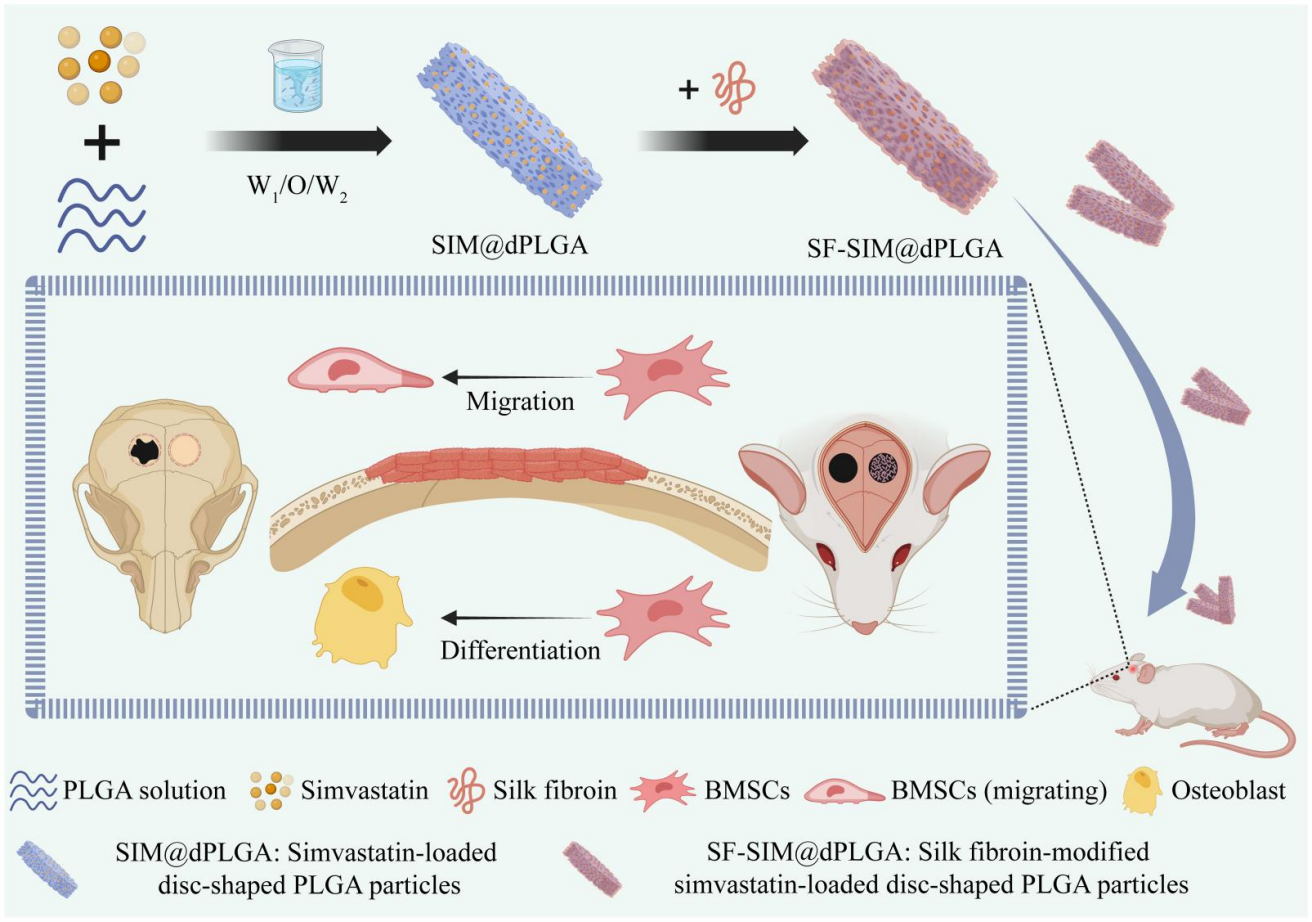
Preparation of SF-SIM@dPLGA for maxillofacial bone regeneration.

In this study, SIM-loaded disc-shaped PLGA particles (SIM@dPLGA) were prepared through the W_1_/O/W_2_ technique according to our previous literature [16]. Subsequently, SF was coated onto the surface of SIM@dPLGA (SF-SIM@dPLGA) via glutaraldehyde cross-linking to prevent SIM dissolution in ethanol, thereby achieving the integrated functions of both osteoconduction and osteoinduction. This design integrates disc-like shape, surface topography, SF surface modification, and sustained SIM release into a microparticle delivery platform, thereby simultaneously enhancing tissue retention/handling and osteogenic bioactivity (Figure 1). The physicochemical features, release profiles, biocompatibility and osteogenic signaling pathways were investigated *in vitro.* The tissue adhesion, procoagulant and maxillofacial bone regeneration were evaluated *in vivo*.

## Experimental methods

### Preparation of SF-SIM@dPLGA

Disc-shaped particles were prepared by a double emulsion-solvent evaporation method (W_1_/O/W_2_) according to our previous study. Briefly, 200 mg PLGA (Mw 50 000, 50:50) (Jinan Daigang Biomaterial, China) and 10 mg SIM (Jiangxi, P.R. China) were dissolved in 4 mL dichloromethane (Fengchuan Chemical Reagent, China) to obtain a polymer solution (O). A 5% mass fraction solution of ammonium bicarbonate (NH_4_HCO_3_) (Aladdin, China) was configured as W_1_, and then 1.25 mL W_1_ was slowly dropped into O, homogenized for 2 min, and poured into 100 mL PVA (Sinopec Sichuan Vinylon Works, China) aqueous solution (W_2_), stirred magnetically (800 rpm) 12 h, rinsed thoroughly with pure water (Millipore Corp, Billerica, USA), and freeze-dried to prepare dPLGA and SIM@dPLGA. Subsequently, SIM@dPLGA was put into 20 mL SF protein solution (4 mg/mL) and mixed with a rotameter for 1 h. After adding 2% glutaraldehyde (Acmec, China) for 0.5 h for cross-linking, the excess aldehyde groups were neutralized with glycine solution (Solarbio, China), washed sufficiently with pure water and freeze-dried to obtain SF-SIM@dPLGA.

### Rat cranial defect model

Animal experiments were approved by the Institute of Radiological Medicine, Chinese Academy of Medical Sciences (IRM-DWLL2021121). Rats (6 weeks old, *n* = 3 per group) were purchased (Spearfish, Beijing). After 1 week of adaptive feeding, all rats were anesthetized with isoflurane gas, and then a longitudinal incision was made along the sagittal line of the skull to fully expose the sagittal suture, bilateral parietal bones and part of the frontal and occipital bones. Two symmetric full-thickness circular calvarial defects (5 mm in diameter) were created on the left and right sides of the skull using a trephine/ring drill. The defects were then filled with different particle formulations according to the group allocation. Finally, the periosteum and skin were sutured sequentially. The body weight of the rats was recorded with a weighing scale.

The methods used for the physical and chemical characterization of the materials, as well as the *in vitro* cell experiments and *in vivo* animal studies, are detailed in the Supplementary material.

## Results and discussion

### Preparation and characterization of SF-SIM@dPLGA

**Figure 2:**
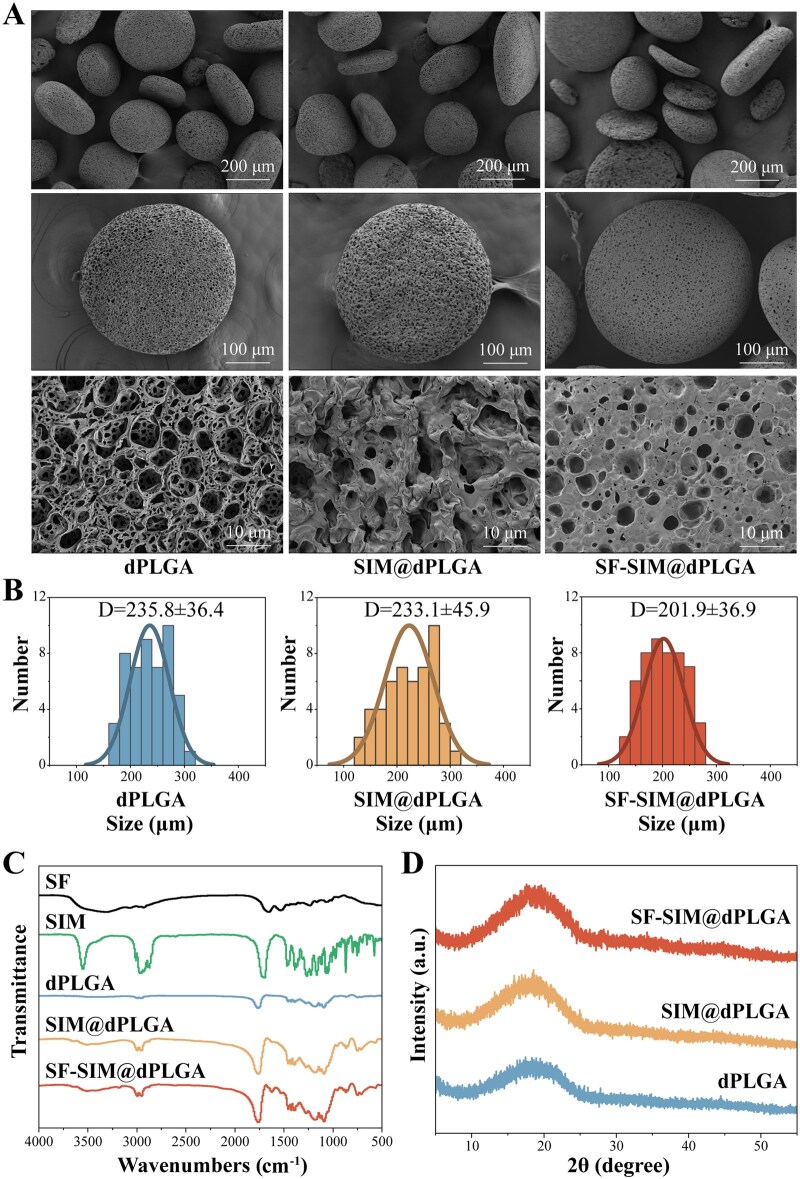
Synthesis and characteristics of SF-SIM@dPLGA. (**A**) SEM images of particles from different groups. Scale bars: 400, 100 and 10 μm, respectively. (**B**) Particle size analysis of particles from different groups (*n* = 50). (**C**) FTIR spectrogram (from top to bottom: SF, SIM, dPLGA, SIM@dPLGA, SF-SIM@dPLGA). (**D**) XRD patterns (from top to bottom: SF-SIM@dPLGA, SIM@dPLGA, dPLGA).

The synthesis of dPLGA and SIM@dPLGA is according to our previous research [16]. Scanning electron microscope (SEM) examinations were performed to determine the successful synthesis of these particles. SEM images showed that dPLGA exhibited a disc-shaped morphology, rough surface and loose porous structure (Figure 2A), which was consistent with our previous results. As demonstrated in our preceding research, the disc-shaped PLGA particles that were prepared via the W_1_/O/W_2_ process in the presence of NH_4_HCO_3_ are believed to originate from the collapse of porous hollow microspheres in an intermediate state. In conditions of elevated foaming agent content and a substantial W_1_/O ratio, a thinner porous shell with diminished mechanical strength is formed. Subsequently, the application of shear stresses during emulsification and solvent evaporation results in the compression of these hollow, porous microspheres into flat, disc-shaped particles. Concurrently, the porous structure undergoes a transformation into a rough surface texture. Consequently, the non-spherical morphology can be regulated through key parameters and homogenized. The unique morphology could increase the surface area of the particles and facilitate cell adhesion [29, 30]. SIM@dPLGA showed the same morphology as dPLGA, indicating that SIM loading did not affect the synthesis and original structure of dPLGA. SF-SIM@dPLGA also displayed a disc-shaped morphology and porous structure, although the surface porosity became slightly smaller. This result suggested that the SF coating via lower concentration organic solution cross-linking did not affect the original structure of the dPLGA, but might lead to particle surface pore size reduction, which also verified the successful modification of SF. Furthermore, the results of the particle size analysis of the three groups indicated that the comparative average particle size was approximately 200 µm (Figure 2B). Fourier transform infrared spectroscopy (FTIR) can be used to further confirm the internal composition of the particles. The infrared characteristic peaks of SIM are O–H at 3550 cm^−1^, CH_2_ and CH_3_ at 2970, 2950 and 2930 cm^−1^, and C=O at 1700 cm^−1^ (Figure 2C). It was observed that SIM@dPLGA and SF-SIM@dPLGA showed significant elevations near the SIM corresponding characteristic peaks. The infrared peaks of SF are located at 1660 and 1540 cm^−1^. Following the coating with SF, the characteristic peaks of SF-SIM@dPLGA were shifted to 1625 cm^−1^ (amide I band) and 1530 cm^−1^ (amide II band), which suggested SIM@dPLGA could be effectively coated with SF. SF underwent a conformational change from α-helical to β-folded, indicating the stability of SF coating was enhanced [31]. Besides, the results of the Energy dispersive spectroscopy (EDS) analysis (Supplementary Figure S1) indicated the presence of protein characteristic N elements on SF-SIM@dPLGA, providing additional evidence of successful encapsulation of SF. The X-ray diffraction (XRD) plots showed no significant crystalline peaks in any of the three sets of particles (Figure 2D, Supplementary Figure S2), indicating the presence of predominantly amorphous characteristics and good internal dispersion of SIM.

**Figure 3:**
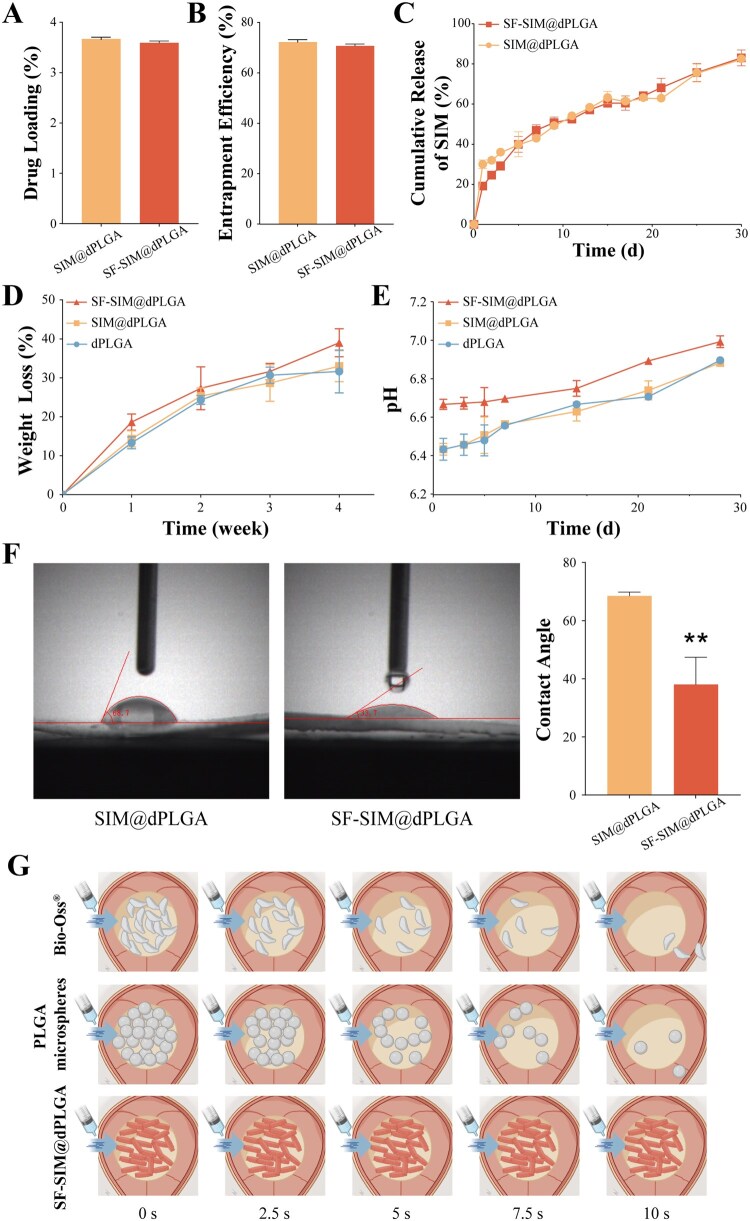
Characteristics of SF-SIM@dPLGA. (**A**) Drug-loading capacity of particles from different groups (*n* = 3). (**B**) Entrapment efficiency of particles from different groups (*n* = 3). (**C**) SIM@dPLGA and SF-SIM@dPLGA release profiles of simvastatin over 30 days (*n* = 3). (**D**) Degradation of particles from different groups (*n* = 3). (**E**) Changes in pH during degradation (*n* = 3). (**F**) Water contact angles for particles from different groups (*n* = 3). (**G**) Experimental schematic diagram of the adhesion ability of different particles to bone tissue. * represents a comparison with CON, with *P* < 0.01 (**).

The drug loading and encapsulation efficiency were similar between the SIM@dPLGA and SF-SIM@dPLGA, with 3.6% for SIM@dPLGA and 3.5% for SF-SIM@dPLGA (Figure 3A and B). These results indicated that, avoiding the use of large amounts of organic solvent, SF coating had little effect on the loading performance of the particles. A release experiment was performed in order to find out whether the SF modification affected the release pattern of the particles. No significant burst release was observed in either group, while SF-SIM@dPLGA showed a slower release profile compared to the SIM@dPLGA in the first week (Figure 3C). Similar to our previous study, SF modification may delay the early drug release. SIM release profiles were fitted (Supplementary Table S1) to several release models (zero-order, first-order, Higuchi, Ritger–Peppas), and both samples were fitted with the Higuchi well. But SF-SIM@dPLGA was best fitted with Ritger–Peppas. Disc-shaped particles as one of the thin polymer slab models, the Fickian diffusion process *n* was 0.50, and for the case-II transport process, *n* was 1.00. Therefore, SIM release of both samples was controlled by Fickian diffusion (*n* = 0.338 ± 0.022; *n* = 0.422 ± 0.013, <0.50), which occurred by the usual molecular diffusion of the drug due to a chemical potential gradient. SIM@dPLGA and SF-SIM@dPLGA showed sustained drug release for 1 month, meeting the need for continuous stimulation at the initial stage of defect repair. Notably, although both particles followed predominantly Fickian diffusion (*n* < 0.5), SF-SIM@dPLGA showed a higher *n* value and a better fit with the Ritger–Peppas model, suggesting that the SF coating introduces an additional interfacial transport resistance and may involve diffusion coupled with coating hydration/relaxation. This coating-regulated release is consistent with the reduced early-stage release within the first week, which may help avoid excessively high local SIM exposure and thereby better support early cell viability/migration during the initial repair phase. Meanwhile, the sustained release over a period of 1 month can provide continuous osteogenic cues that are beneficial for subsequent differentiation, mineralization, and longer-term regeneration. As depicted in Figure 3D, the *in vitro* degradation results demonstrated that both SIM@dPLGA and SF-SIM@dPLGA possessed good biodegradability. It is widely acknowledged that the PLGA particles can fully degrade into carbon dioxide and water; however, the acidic by-products may lead to local inflammatory reactions [32]. To ascertain whether SF modification can mitigate the aforementioned shortcomings, the pH value was measured during the degradation process. The results indicated that although all three degradation products were weakly acidic, the pH value of SF-SIM@dPLGA was slightly higher than that of dPLGA and SIM@dPLGA (Figure 3E). SF coating could help to reduce the decrease in microenvironment pH caused by the local accumulation of acidic products, such as lactic acid and glycolic acid, during PLGA degradation. SF, as the outermost hydrophilic coating, hydrates and loosens early in degradation, altering the particles’ overall degradation and interfacial acid enrichment. Its improved wettability enhances water exchange, promoting outward diffusion and dilution of acidic degradation products, consistent with prior findings that increased polymer hydrophilicity speeds acid release into the medium [26, 33]. We conducted water contact angle tests to evaluate whether the hydrophilicity of SF-SIM@dPLGA was genuinely enhanced before and after modification with SF protein. The addition of SF to SIM@dPLGA resulted in a decrease in the water contact angle (Figure 3F), indicating the improvement of hydrophilicity in SF-SIM@dPLGA. The preceding experiments demonstrated that SF-SIM@dPLGA could be successfully synthesized through the double emulsion-solvent evaporation method and modified with chemical cross-linking. The prepared SF-SIM@dPLGA displayed disc-shaped morphology, highly efficient drug-carrying capacity, improved drug release property, reduced acidic by-products, and increased hydrophilicity, suggesting an ideal bone substitute for bone regeneration. According to our previous studies, the preparation of dPLGA in this study is based on the conventional W_1_/O/W_2_ double emulsion-solvent evaporation process, which is widely adopted in pharmaceutical manufacturing and inherently scalable [34]. By controlling the key process parameters such as polymer concentration, phase ratio, emulsification energy and solvent removal rate, high yield and high reproducibility of dPLGA are expected to be commercially produced. The SF coating step is equally suitable for large-scale production and operates on the principles of surface adsorption followed by cross-linking reactions [16, 21]. Reproducible coating can be achieved by regulating SF concentration, coating time and cross-linking conditions, and by carrying out quality validation. While the current study was conducted on a laboratory scale, the characteristics of this process indicate that both the preparation of the particles and the SF functionalization can be scaled up through appropriate process validation.

In order to further evaluate the potential of the particles for *in vivo* applications, where resistance to bodily fluid erosion is crucial, a dynamic erosion assay was conducted. PLGA spherical microspheres, commercial Bio-Oss^®^ bone powder, and our SF-SIM@dPLGA particles were subjected to a fluid flow for 10 seconds in order to simulate the initial post-implantation fluid erosion environment. As illustrated in Figure 3G and Supplementary Figure S3, the spherical PLGA microspheres and Bio-Oss^®^ particles were almost entirely eliminated within this brief period. Conversely, the SF-SIM@dPLGA particles demonstrated markedly enhanced retention, with the majority of particles maintaining adhesion. The remarkable anti-washing property exhibited by dPLGA can be attributed to its unique disc-shaped morphology and coarse surface topography, which provide a larger contact area and enhance mechanical interlocking with the underlying substrate. Furthermore, the SF coating improved hydrophilicity (Figure 3F), which may further facilitate intimate contact with the moist tissue surface. This superior retention capability suggests that SF-SIM@dPLGA is more likely to be retained at the bone defect site, providing a stable and long-lasting platform for osteoconduction and drug delivery, which is a significant advantage over conventional spherical particles or commercial bone grafts. The majority of existing SIM delivery strategies rely on the embedding of polymer microspheres within scaffolds or injectable reservoirs to mitigate burst release and prolong effective exposure time, often employing carrier-assisted positioning techniques, such as sol–gel transformation hydrogels, which achieve retention in complex anatomical sites [35, 36]. In contrast, SF-SIM@dPLGA, in this study, adopts a large size disc-based structure that optimizes release behavior through interfacial modification. The application scope of this system is similar to that of the most commonly used bone powder material in clinical applications. The geometry of their discs and the roughness of their surfaces have been demonstrated to promote stable cell/particle interactions and rapid blood clot integration. This suggests the potential for local retention without the necessity of prefabricated scaffolds. However, integration of the particles into clinically compatible carriers during clinical translation could further enhance usability and reduce particle dispersion during delivery.

### SF-SIM@dPLGA promotes cell adhesion and migration

**Figure 4:**
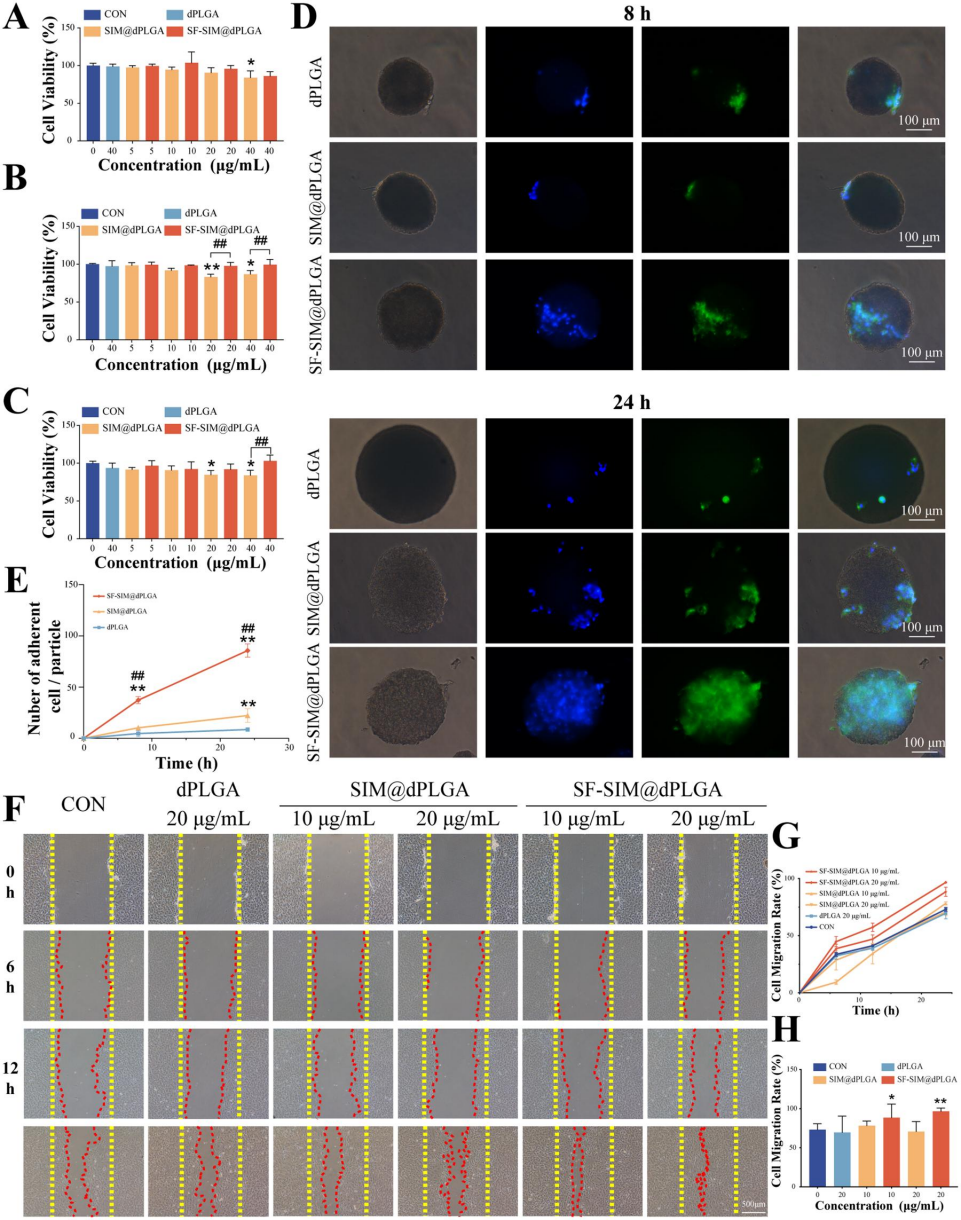
Biocompatibility and cell adhesion of SF-SIM@dPLGA. (**A**) Cell viability with different particles for 24 h. (**B**) Cell viability with different particles for 48 h. (**C**) Cell viability with different particles for 72 h. (**D**) Cell adhesion of BMSCs on different particles at 8 and 24 h. (**E**) Quantitative statistics of BMSCs on different particles. (**F**) Wound-healing images. Scale bar: 500 μm. (**G**) Statistical analysis of wound-healing test results. (**H**) Statistical analysis of wound-healing test results at 24 h. * represents a comparison with CON, with *P* < 0.05 (*) and *P* < 0.01 (**). ^#^ represents a comparison between the labeled two groups, with *P* < 0.01 (^##^).

To evaluate the biocompatibility of free SIM, dPLGA, SIM@dPLGA and SF-SIM@dPLGA, bone mesenchymal stem cells (BMSCs) incubated with different particles were detected through CCK-8 assay. The results showed that 2-µM SIM did not affect the cell viability of BMSCs, while 5-µM SIM could significantly inhibit the cell viability of BMSCs (Supplementary Figure S4), reminding us that the SIM concentration should be controlled below 2 µM. It has been demonstrated that the optimal concentration of SIM to promote cellular osteogenesis toward differentiation is approximately 1 µM [37]. Consequently, considering the SIM cytotoxicity test results in conjunction with the above statement, the concentration of SIM should be maintained at 1–2 µM. According to the loading capacity of SIM, the concentrations of SIM@dPLGA and SF-SIM@dPLGA were adjusted to be approximately 10 and 20 µg/mL. The CCK-8 experiment was then extended to encompass both sides of this concentration range, and dPLGA was set to the highest concentration of 40 µg/mL. Cell viability results showed that 40-µg/mL dPLGA did not affect the cell viability of BMSCs after 24–72 h incubation (Figure 4A–C). SIM@dPLGA (5 and 10 µg/mL) could not influence the cell viability of BMSCs during 72-hour incubation, while a relative decrease in cellular activity was observed in the higher concentration of SIM@dPLGA group. However, SF-SIM@dPLGA in the concentration from 5 to 40 µg/mL did not show significant cytotoxicity, indicating that SF-SIM@dPLGA possessed excellent biocompatibility. To further examine the cell adhesion of different particles, BMSCs were incubated with dPLGA, SIM@dPLGA, or SF-SIM@dPLGA for 8 and 24 h. The results displayed that BMSCs could adhere to the surface of these particles at 8 h, and the number of cells on the SF-SIM@dPLGA was more than that of the other two groups. After 24 h of incubation, the number of cells on the surface of all particles increased, with the surface of SF-SIM@dPLGA almost crawling with cells and showed much more cell adhesion compared with dPLGA and SIM@dPLGA groups (Figure 4D). Since the particle sizes were similar, we quantified adhesion by counting the number of cells adhering to the surfaces of the particles across multiple random fields of view at each time point. As illustrated in Figure 4E, cell adhesion to SF-SIM@dPLGA particles was significantly higher than to SIM@dPLGA and dPLGA particles at both the 8- and 24-h time points (*P* < 0.01). These results suggested that SF modification could significantly promote the cell adhesion capacity of dPLGA. The hydroxyl group of serine in SF can form hydrogen bonds with receptors or proteins on cell membranes, thereby promoting cell adhesion [38, 39]. All these results verified that SF-SIM@dPLGA showed superior biocompatibility and cellular adhesion capacity.

Cell migration was then detected through the scratch assay. As shown in Figure 4F, groups of dPLGA, SIM@dPLGA and 10-μg/mL SF-SIM@dPLGA showed no obvious difference with the control group after 6 h of treatment, while the 20-μg/mL SF-SIM@dPLGA group showed decreased defect area. After 12 h of incubation, the defect area in the SF-SIM@dPLGA groups began to shrink, with the smallest remaining defect area observed in the 20 μg/mL SF-SIM@dPLGA group. And after 24 h of incubation, the defect areas of the SF-SIM@dPLGA groups with different concentrations were significantly reduced, among which the remaining defect area of the 20-μg/mL SF-SIM@dPLGA group almost disappeared. Whereas the SIM@dPLGA and dPLGA groups displayed no significant difference with the control group. Quantitative analysis showed that SF-SIM@dPLGA could significantly promote the cell migration of BMSCs, while dPLGA and SIM@dPLGA groups showed no significant difference with the control group (Figure 4G and H). SF proteins have been demonstrated to facilitate cell migration by inducing the expression of factors such as cell cycle proteins and fibronectin [40]. As evidenced by the aforementioned findings, the SIM@dPLGA groups were unable to facilitate cell migration, which may be attributed to the stage-dependent bioactivity of SIM. Recent studies have suggested that BMSC recruitment and subsequent osteogenic differentiation rely on distinct effective dose windows and spatiotemporal drug exposure profiles, which may not be simultaneously achieved under a single release condition [41, 42]. For this purpose, we additionally made Transwell assays to independently validate the effect of SIM concentration on cell migration (Supplementary Figure S5). The results showed that SIM promotes the migration of rBMSCs in a concentration-dependent yet non-linear manner, with the 0.1-μM concentration inducing the strongest chemotactic response. This response curve suggests that migration and osteogenic differentiation may require distinct exposure thresholds. Lower SIM concentrations may act as a chemotactic stimulate to enhance cell recruitment, whereas higher concentrations may drive cells to differentiation programmes. Because this study mainly focuses on osteogenic research, the concentration was chosen to be 1 μM. Furthermore, concentrations exceeding 2 μM exhibited cytotoxicity in our experiments, highlighting the importance of maintaining SIM within an appropriate therapeutic range for practical applications. In contrast, the SF-SIM@dPLGA could promote cell migration, suggesting that SF proteins may play a pivotal role in promoting cell migration.

### SF-SIM@dPLGA promotes osteogenesis *in vitro*

**Figure 5:**
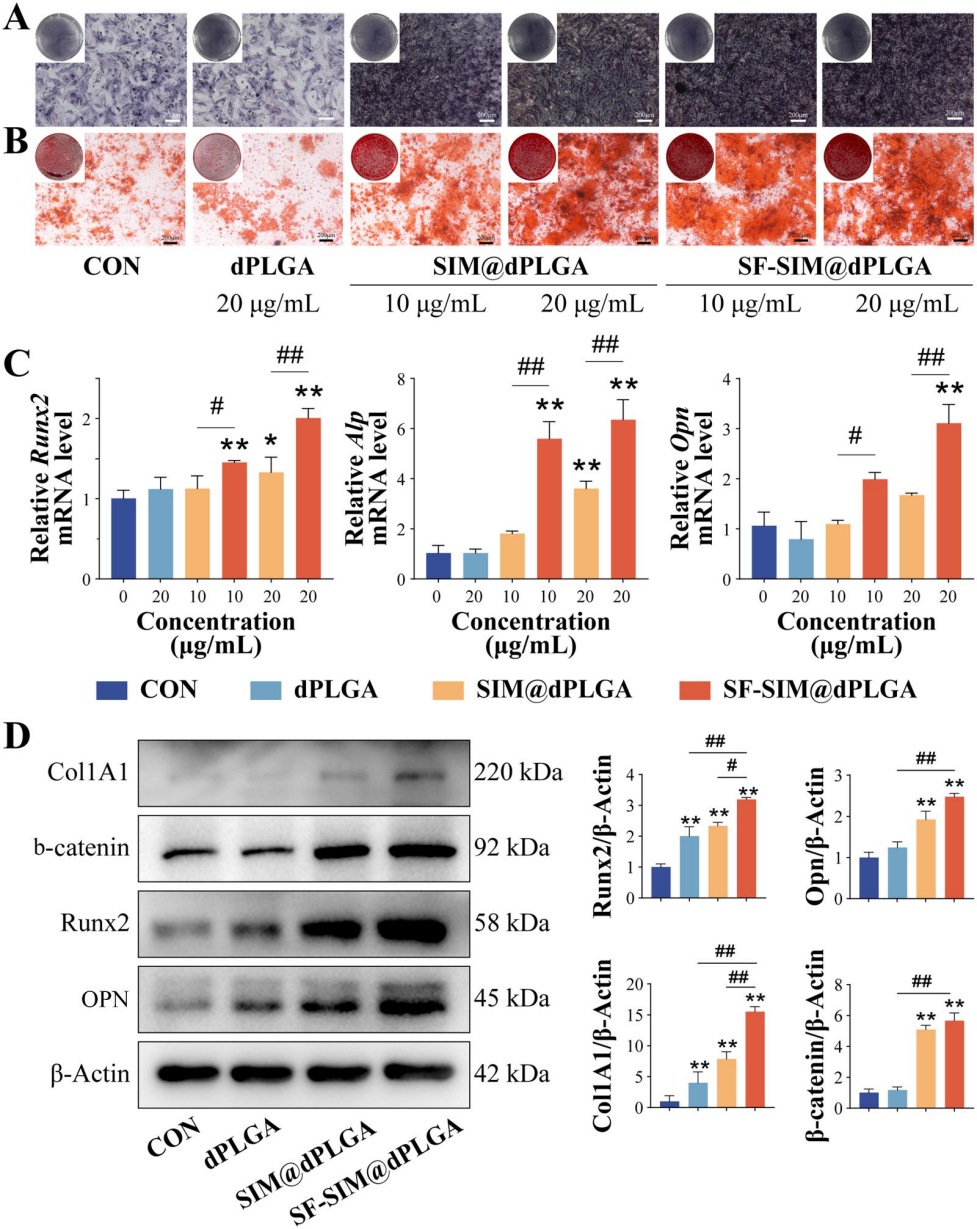
SF-SIM@dPLGA promotes osteogenic differentiation of BMSCs. (**A**) ALP staining images. Scale bar: 200 μm. (**B**) ARS images. Scale bar: 200 μm. (**C**) Gene expression of *Runx2*, *Alp* and *Opn*. (**D**) Protein expression of Col1A1, Runx2, OPN and β-catenin. The concentrations of the subgroups were all 20 μg/mL. * represents a comparison with the control group, with *P* < 0.05 (*) and *P* < 0.01 (**). ^#^ represents a comparison between the labeled two groups, with *P* < 0.05 (^#^) and *P* < 0.01 (^##^).

Osteogenic differentiation of BMSCs is of the utmost importance in bone regeneration. To explore the effects of SF-SIM@dPLGA on the osteogenic differentiation of BMSCs, alkaline phosphatase (ALP) staining and alizarin red S (ARS) staining were employed. The results of ALP staining demonstrated that there was no significant difference between the dPLGA-treated BMSCs and the control group (Figure 5A). However, BMSCs treated with SIM@dPLGA and SF-SIM@dPLGA exhibited elevated ALP activity. Furthermore, SF-SIM@dPLGA treatment demonstrated deeper staining, indicating that SF-SIM@dPLGA could enhance the early osteogenic differentiation of BMSCs. ARS staining results showed the same trends as ALP staining (Figure 5B). Both SIM@dPLGA and SF-SIM@dPLGA groups exhibited increased deposition of red calcium nodules, and the SF-SIM@dPLGA groups, especially at high concentration of the SF-SIM@dPLGA group showed the deepest staining, suggesting that SF-SIM@dPLGA could promote the mineralization process. In parallel, the levels of osteogenic markers, such as Runt-related transcription factor 2 (Runx2), alkaline phosphatase (ALP), osteopontin (OPN), and collagen type I alpha 1 (Col1A1), were detected by quantitative real-time reverse transcription polymerase chain reaction (qRT-PCR) and western blot. qRT-PCR results showed that both SIM@dPLGA and SF-SIM@dPLGA were capable of upregulating the translation levels of *Runx2*, *Alp*, and *Opn*, and high concentrations of SF-SIM@dPLGA could significantly increase the translation levels of these markers (Figure 5C). The protein levels of Runx2, OPN and Col1A1 detected by western blot and semi-quantitative analysis were consistent with the qRT-PCR analyses (Figure 5D). SF-SIM@dPLGA could significantly promote the expression levels of Runx2, OPN and Col1A1, demonstrating that SF-SIM@dPLGA could effectively promote the osteogenic differentiation of BMSCs by enhancing the expression of osteogenic markers. According to previous researches, both SIM and SF could promote the osteogenesis of BMSCs [43–45]. Our results also demonstrated that SF-SIM@dPLGA possessed an excellent promotion effect on osteogenic differentiation of BMSCs, providing a good basis for bone regeneration.

Deeper insights into the mechanisms underlying the osteogenic differentiation of BMSCs revealed that SIM and SF promote osteogenic differentiation through the Wnt/β-catenin pathway [46–48]. The Wnt/β-catenin pathway is a crucial signaling pathway implicated in a multitude of cellular processes, including cell proliferation, differentiation, migration and apoptosis, especially osteogenesis of stem cells [49–51]. Therefore, the expression level of β-catenin was also detected. The results showed that both SIM@dPLGA and SF-SIM@dPLGA could promote the expression of β-catenin, and the upregulation of β-catenin protein was most significant in the SF-SIM@dPLGA group (Figure 5D). The elevated β-catenin expression suggests that Wnt/β-catenin signaling may be involved in the osteogenic response induced by SF-SIM@dPLGA; however, further functional studies are required to establish causality. In order to further investigate the mechanism underlying the effects of the particles, future studies will employ Wnt/β-catenin inhibition or gene silencing techniques to verify whether this pathway is essential for the SF-SIM@dPLGA-induced enhancement of osteogenesis.

### SF-SIM@dPLGA promotes bone regeneration *in vivo*

**Figure 6:**
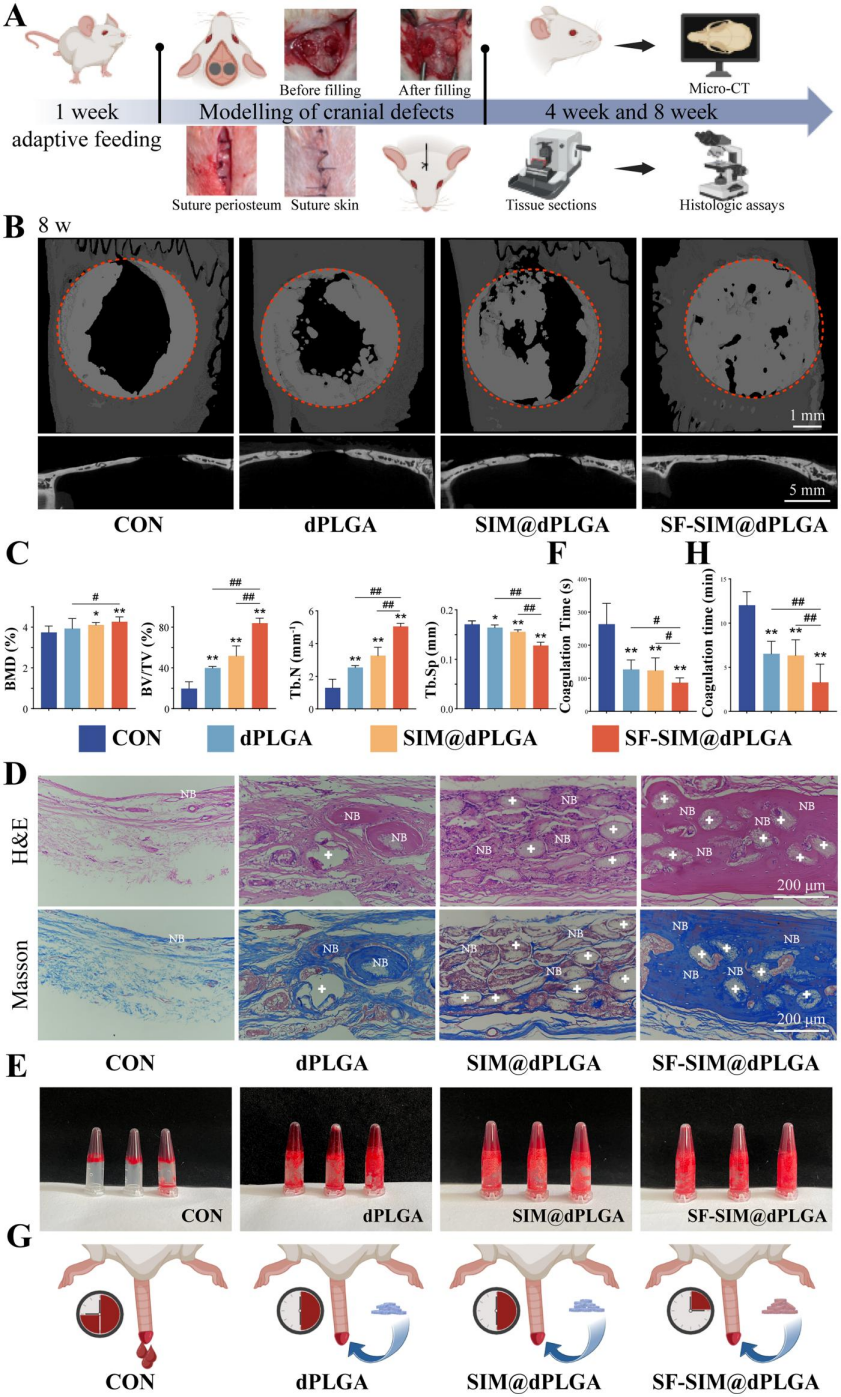
SF-SIM@dPLGA promotes bone regeneration *in vivo* after 8 weeks. (**A**) Flowchart of rat cranial defect modeling process. (**B**) Micro-CT images at 8 weeks. Scales: 1 and 5 mm, respectively. (**C**) BMD, BV/TV, Tb. N and Tb. Sp analysis. (**D**) H&E and Masson trichrome staining images. + indicates the location of residual discoidal particles, HB indicates host bone (HB), and NB indicates newly formed bone (NB). (**E**) Plasma recalcification experiments results. (**F**) Quantitative analysis of plasma recalcification time. (**G**) Schematic diagram of tail cutting hemostasis experiment. (**H**) Quantitative analysis of coagulation time during tail-breaking hemostasis experiment. * represents a comparison with the control group, with *P* < 0.05 (*) and *P* < 0.01 (**). ^#^ represents a comparison between the labeled two groups, with *P* < 0.05 (^#^) and *P* < 0.01 (^##^).

To investigate the effects of SF-SIM@dPLGA on bone regeneration, a rat cranial defect model was constructed [52] (Figure 6A). Bone regeneration progress was assessed via micro-CT scans and histochemical stains at 4 and 8 weeks post-implantation. After 4 weeks of healing, the micro-CT results showed that there was minimal new bone growth in the control group, while the SF-SIM@dPLGA group exhibited an obvious increase in new bone formation (Supplementary Figure S6A). Quantitative analysis showed the same results. Bone mineral density (BMD) and bone volume fraction (BV/TV) represented the extent of new bone formation, and SF-SIM@dPLGA could significantly increase the BMD and BV/TV (Supplementary Figure S6B). Tb. N and Tb. Sp represent the number of trabeculae and trabecular separation. The results showed that SF-SIM@dPLGA treatment could significantly increase Tb. N and decrease Tb. Sp in the newly formed bone tissues, indicating that the bone was compact. Furthermore, histological staining was also employed to evaluate the newly formed bone tissue. H&E staining revealed that only a minimal amount of sparse fibrous tissue was formed in the control group, while the SF-SIM@dPLGA group displayed a higher prevalence of newborn bone tissue compared to the area of bone defects (Supplementary Figure S6C). Masson staining demonstrated the presence of more mature collagen fibers (blue-stained) in the SF-SIM@dPLGA group compared to other groups (Supplementary Figure S6C), indicating a higher degree of maturity. The aforementioned results suggest that SF-SIM@dPLGA possessed a distinctive advantage in the promotion of bone regeneration *in vivo*, encompassing not only the largest area of new bone formation but also a more expeditious progression of bone regeneration and the development of more mature bone tissue.

After 8 weeks of healing, the micro-CT results showed that there was a minimal area of new bone formation in the control group; bone defect areas were obviously decreased in the dPLGA and SIM@dPLGA groups, whereas the bone defects were almost completely repaired in the SF-SIM@dPLGA group (Figure 6B). Quantitative analysis results confirmed that BMD, BV/TV and Tb. N of the SF-SIM@dPLGA group were significantly higher than those of the other three groups, Tb. Sp was lower (Figure 6C), indicating that SF-SIM@dPLGA could effectively promote bone regeneration and accelerate the osseous maturation. H&E and Masson staining revealed that the SF-SIM@dPLGA group exhibited more compact and mature calcified lamellar bone than the other three groups (Figure 6D). Compared to the 4-week staining images, the SF-SIM@dPLGA particles presented in the defect were gradually degraded over time. Meanwhile, the newly formed bone was observed in the vicinity of incompletely degraded particles, suggesting that the new bone growth occurred around the SF-SIM@dPLGA particles and continued to grow inward as the particles degraded. These results suggest that SF-SIM@dPLGA can effectively promote bone regeneration *in vivo* by promoting new bone formation and maturity. In addition to osteogenic induction, increased evidence suggests that the shape and surface topography of biomaterials can modulate the local osteoimmune microenvironment, particularly macrophage polarization (M1/M2 phenotype), which plays a critical role in bone regeneration [53, 54]. In consideration of the disc-like morphology and rough surface features of the microparticles, it can be hypothesized that SF-SIM@dPLGA may influence early immune responses at the defect site and thereby contribute to a more favorable regenerative microenvironment. Therefore, future research on the material itself regarding the immunoregulatory mechanisms related to bone formation will be of great significance.

### SF-SIM@dPLGA possesses excellent procoagulant capacity and biocompatibility *in vivo*

In order to verify the procoagulant and tissue adhesion effects of SF-SIM@dPLGA, a plasma recalcification time assay and a rat tail amputation assay were conducted. The results of the plasma recalcification time assay demonstrated that all three particles exhibited a notable coagulation effect (Figure 6E and F), thereby substantiating the hypothesis that the disc-shaped particles possess the capacity to accelerate coagulation. This is achieved through the formation of blood clots at a faster rate, which is a consequence of the particles’ ability to absorb water and undergo coagulation. Among the particles, SF-SIM@dPLGA exhibited the shortest coagulation time, suggesting that the incorporation of SF may augment the coagulation efficacy. This is potentially attributable to SF’s capacity to enhance water absorption, thereby accelerating blood coagulation and enhancing hemostatic efficiency [55]. In the rat tail-break model, SF-SIM@dPLGA demonstrated a greater propensity to be adsorbed and form a larger clot around the wound, with the shortest hemostasis time (Figure 6G and H and Supplementary Figure S7). The disc-shaped particles increase the surface area of the particles, ensuring a larger contact area with the tissue. The addition of SF has the potential to significantly enhance the tissue adhesion of the particles, making SF-SIM@dPLGA ideal for hemostasis and healing promotion [56]. The specific morphology and SF modifications may facilitate the formation of blood clots at the site of injury, thereby accelerating the tissue healing process. The enhanced coagulation-promoting effect of SF-SIM@dPLGA represents a potentially viable strategy for bone regeneration. The above experiments demonstrate that SF-SIM@dPLGA exhibits excellent tissue adhesion and rapid hemostatic integration capabilities, which facilitates their local retention. They are particularly well suited to moist surgical environments. In clinical applications, combining the microparticles with hydrogels or gel sponges optimizes delivery efficiency and improves operability. This may enhance the microparticles’ retention capacity in defect sites, especially in irregular or hemorrhagic areas.

The biocompatibility of SF-SIM@dPLGA was detected by the hemolysis method and *in vivo* experiments. dPLGA, SIM@dPLGA and SF-SIM@dPLGA were non-hemolytic, and the hemolysis rates were significantly lower than the control group, suggesting that all particles had no adverse effects on red blood cells and were suitable as a blood contact transplantation material (Supplementary Figure S8). The *in vivo* biocompatibility of SF-SIM@dPLGA was detected during the healing process of bone defects. Body weight of rats revealed that all groups displayed no significant weight loss (Supplementary Figure S9). H&E staining of important organs was further excised to determine the *in vivo* toxicity of all particles. The results showed that brain, heart, spleen, liver, lung and kidney tissues in all groups exhibited no obvious difference in tissue structure and cell morphology (Supplementary Figure S10A), indicating that dPLGA, SIM@dPLGA and SF-SIM@dPLGA did not affect important organs during the whole therapeutic process. Furthermore, the peripheral blood routine and blood biochemistry were also employed. The results showed that all groups displayed no obvious abnormalities (Supplementary Figure S10B and C). All of these results indicated that SF-SIM@dPLGA possessed excellent biocompatibility *in vivo*.

## Conclusion

In summary, non-spherical drug delivery vehicle was optimized in particle shape, surface topography, SF and SIM activity. The fabricated platform, namely SF-SIM@dPLGA, could be efficiently synthesized through the double emulsion-solvent evaporation method and surface modification. The complex exhibited favorable cell/tissue adhesion and sustained drug release ability as well as favorable biocompatibility and appropriate degradability, which endow the system with dual functionality of osteoconduction and osteoinduction. The surface morphology of dPLGA, characterized by reduced curvature and increased roughness, creates a favorable environment for cell growth. Additionally, SF modification improves cell/tissue adhesion and migration while mitigating acid degradation, and SIM facilitates osteogenic differentiation. All these capacities collectively enabled SF-SIM@dPLGA to effectively facilitate the formation of blood clots, promote cell homing and differentiation, and finally accelerate the bone regeneration process. Consequently, SF-SIM@dPLGA holds great promise as a novel therapeutic approach for bone regeneration.

## Supplementary Material

rbag053_Supplementary_Data
