# Supplementary material for: Silk-coated PLGA non-spherical microparticles for simvastatin delivery in rat maxillofacial bone regeneration
Source: Regen Biomater. 2026 Mar 10;13:rbag053. doi: 10.1093/rb/rbag053 (PMC13082902; doi:10.1093/rb/rbag053)
Supplement: rbag053_Supplementary_Data [file rbag053_supplementary_data.docx]

#### Supporting material

**Silk-coated PLGA** **non-spherical microparticles for simvastatin delivery in rat maxillofacial bone regeneration**

Junjiang Zhang^1, 2, #^, Yuqi Chang^1, 2, #^, Yiwen Zhou^1, 2^, Yang Zhou^1, 2^, Jiayin Li^1, 2^, Feng Wang^2, 3^, Zhimin Zhou^4,^ *, Lei Sui^1, 2,^ *, Yanjing Li^1, 2,^ *

Table S1. Release models of SIM-loaded PLGA dics-shape microshpheres without or with silk coating

|  | SIM@dPLGA | SF-SIM@dPLGA |
| --- | --- | --- |
| Zero order | y=30.90+1.75x | y=22.28+2.40x |
|  | R^2^=0.97164 | R^2^=0.92819 |
| First order | y=65.40*(1-e^-0.21^) | y=64.13*(1-e^-0.20^) |
|  | R^2^=0.71577 | R^2^=0.91172 |
| Higuchi | y=11.28x^1/2^+15.53 | y=14.02x^1/2^+5.73 |
|  | R^2^=0.97502 | R^2^=0.99073 |
| Peppas | y=23.93x^0.34^ | y=19.15x^0.42^ |
|  | R^2^=0.96 | R^2^=0.99284 |


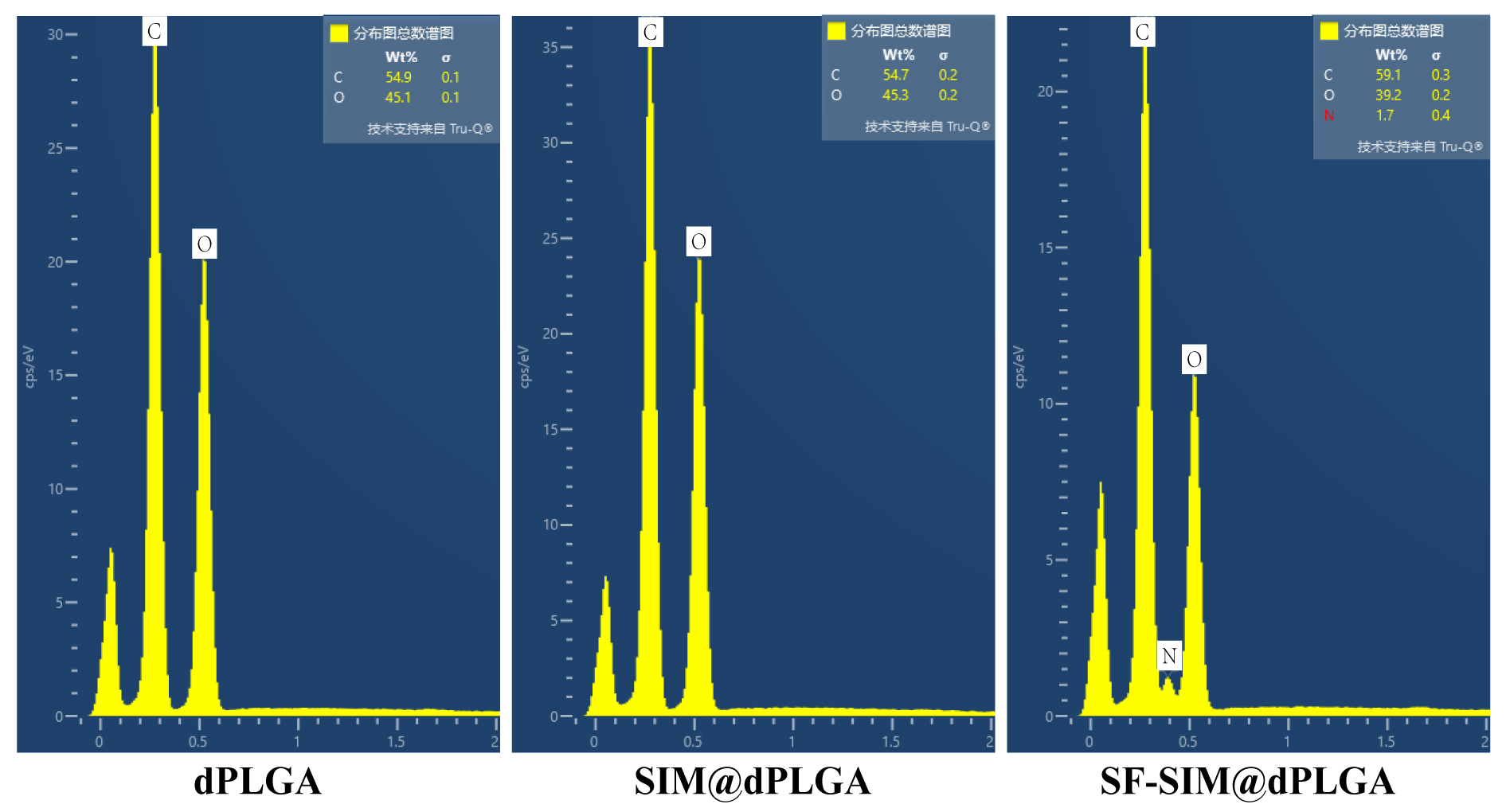


Figure S1. EDS energy spectral analysis of dPLGA,SIM@dPLGA, SF-SIM@dPLGA.


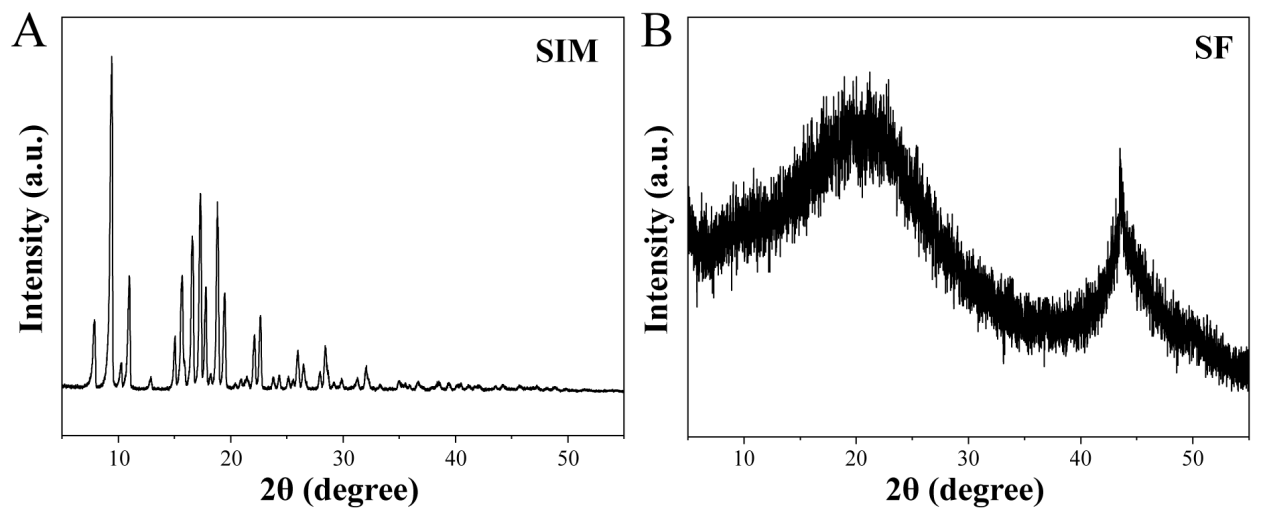


Figure S2. (A) XRD pattern of SIM; (B) XRD pattern of SF.


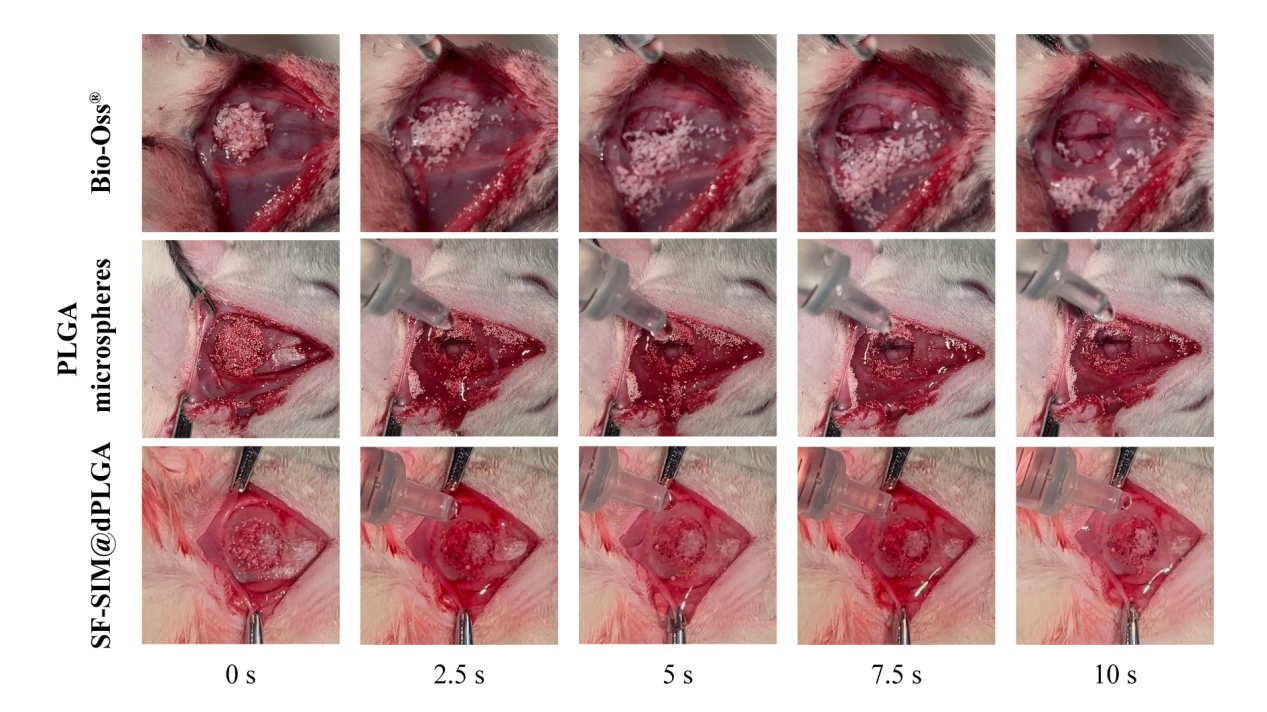


Figure S3. Adhesion capabilities of bone tissues with different particles.


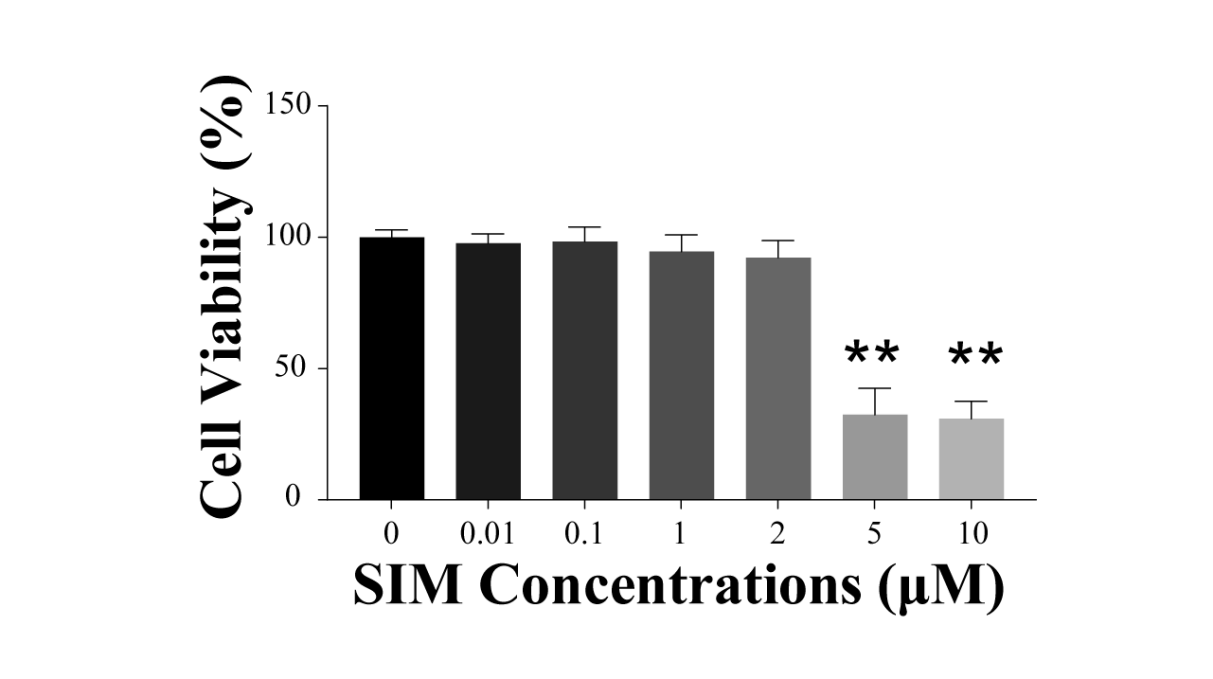


Figure S4. Cell viability with different concentrations of SIM for 24 hours.


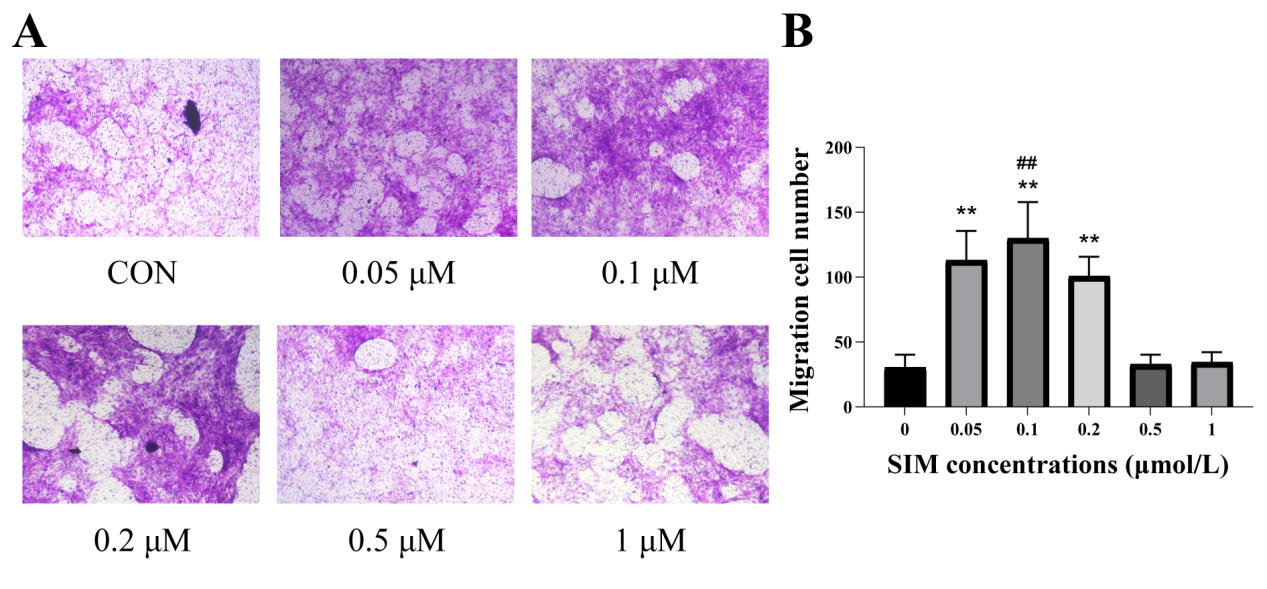


Figure S5. The effect of simvastatin (SIM) concentration on rBMSCs migration was assessed using a Transwell assay. (A) Migration images for each group; (B) Number of cells migrating in each group. ** represents p < 0.01 vs. 0 μM; ## represents p < 0.01 vs. 0.05 μM.


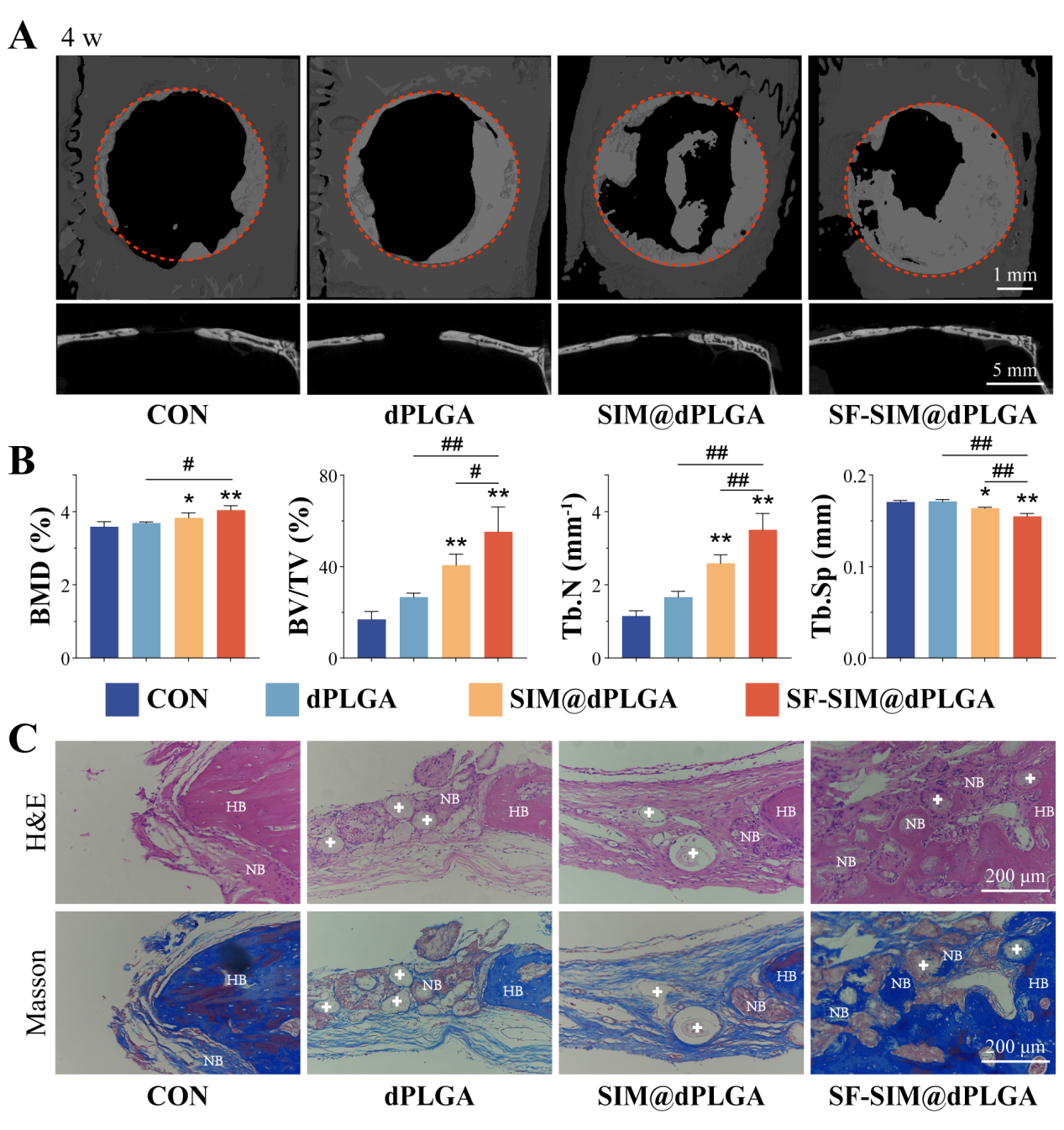


Figure S6. SF-SIM@dPLGA promotes bone regeneration in vivo after 4 weeks. (A) Micro-CT images at 4 weeks. Scales: 1mm and 5mm respectively; (B) BMD, BV/TV, Tb.N and Tb.Sp analysis; (C) H&E and Masson trichrome staining images. + indicates the location of residual discoidal particles, HB indicates host bone (HB), and NB indicates newly formed bone (NB). * represents a comparison with the control group, with P < 0.05 (*) and P < 0.01 (**). # represents a comparison between the labeled two groups, with P < 0.05 (#) and P < 0.01 (##).


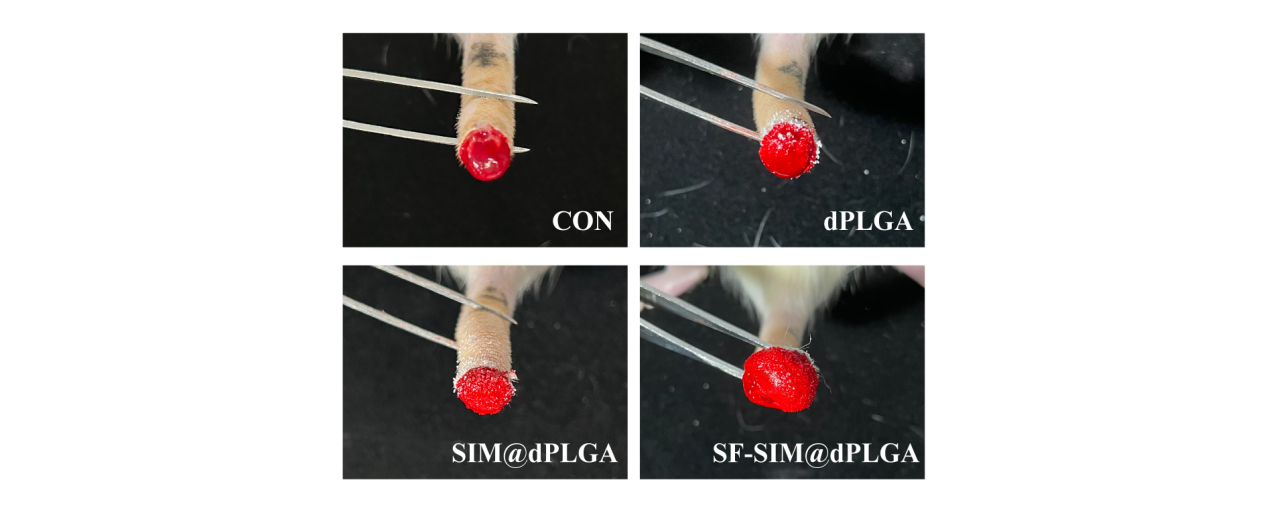


Figure S7. Tail-breaking haemostasis experiment results.


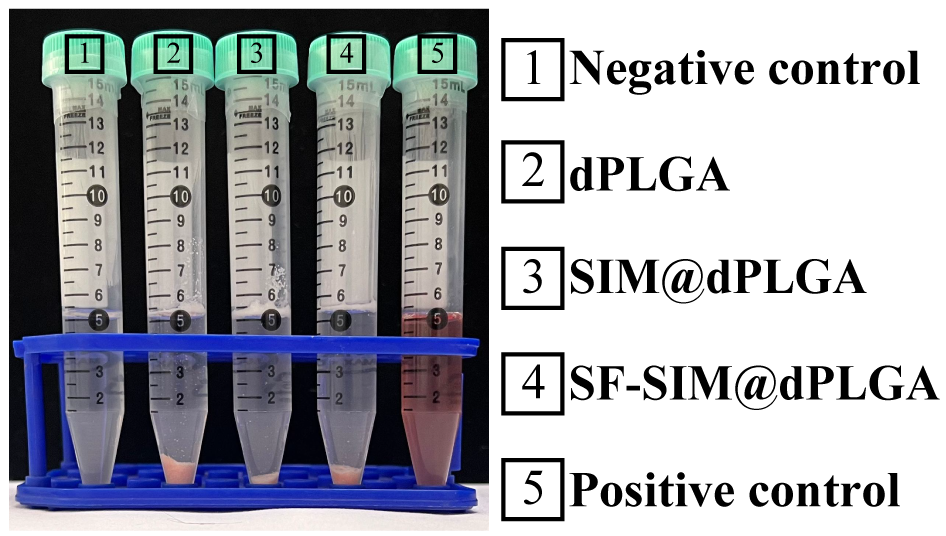


Figure S8. Haemolysis test results.


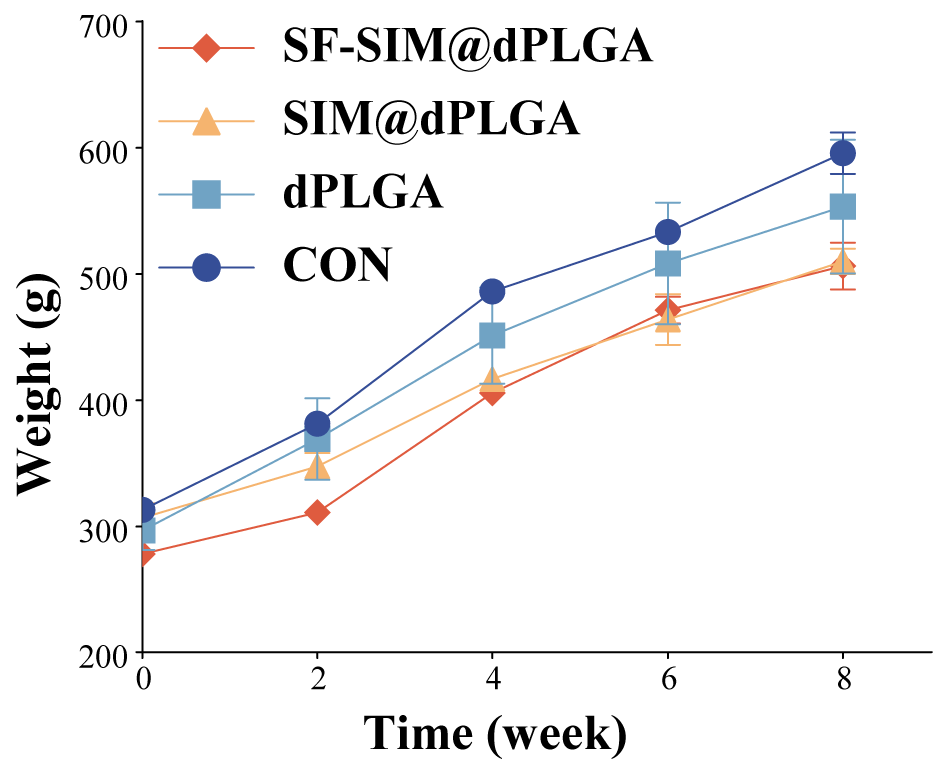


Figure S9. Changes in body weight of rats.


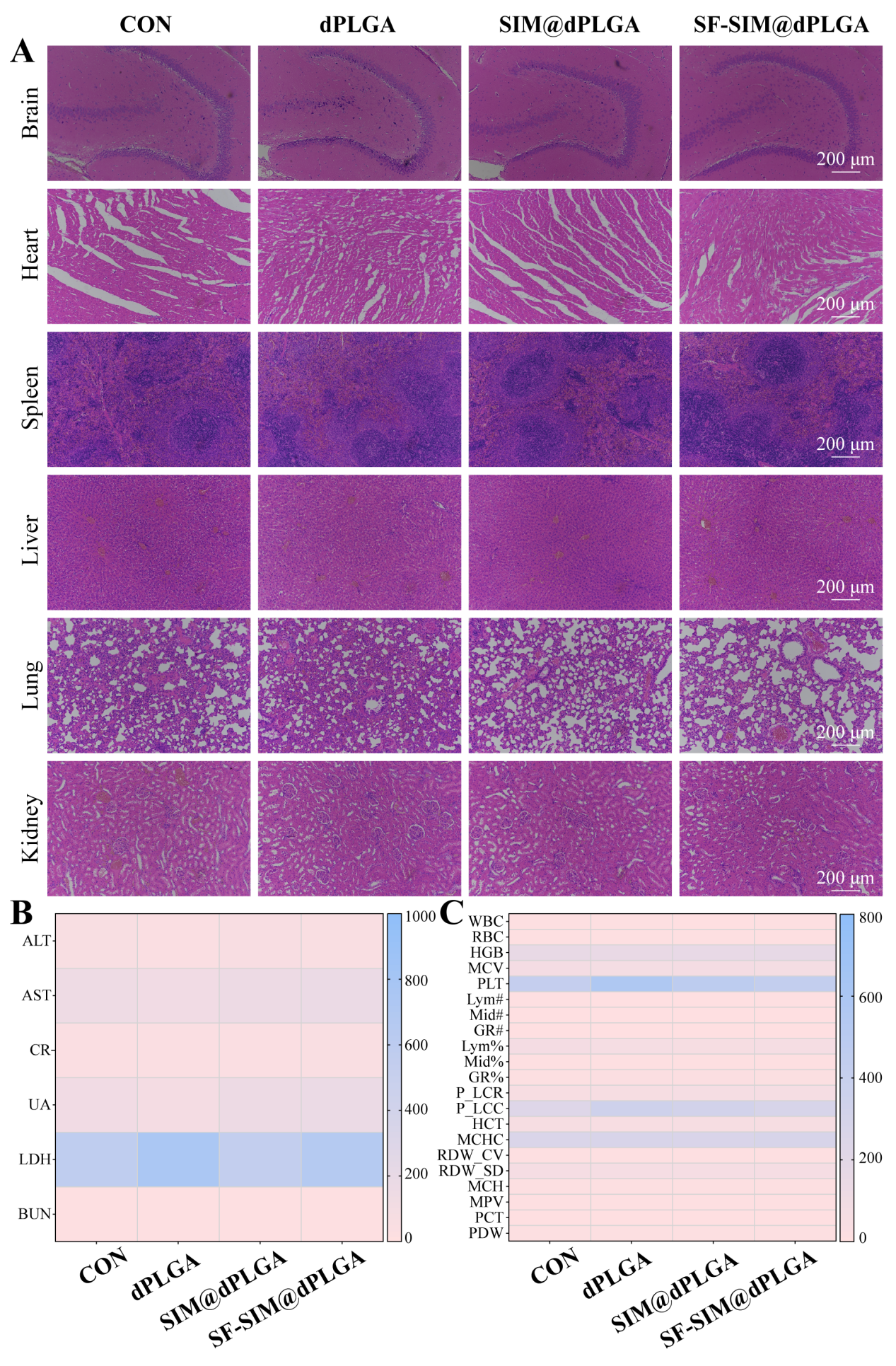


Figure S10. In vivo biocompatibility of SF-SIM@dPLGA. (A) H&E staining images of major organs (brain, heart, spleen, liver, lung, kidney). Scale bar: 200 μm; (B) Blood biochemistry result; (C) Blood routine result.

**Experimental methods**

**Preparation of SF-SIM@dPLGA**

Disc-shaped particles were prepared by a double emulsion-solvent evaporation method (W_1_/O/W_2_) according to our previous study. Briefly, 200 mg PLGA (Mw. 50,000, 50 : 50) (Jinan Daigang Biomaterial, China) and 10 mg SIM (Jiangxi, P. R, China) were dissolved into 4 mL dichloromethane (Fengchuan Chemical Reagent, China) to obtain a polymer solution (O). A 5 percent mass fraction solution of ammonium bicarbonate (NH_4_HCO_3_) (Aladdin, China) was configured as W_1_, and then 1.25 mL W_1_ was slowly dropped into O, homogenized for 2 min, and poured into 100 mL PVA (Sinopec Sichuan Vinylon Works, China) aqueous solution (W_2_), stirred magnetically (800 rpm) 12 h, rinsed thoroughly with pure water (Millipore Corp, Billerica, USA), and freeze-dried to prepare dPLGA and SIM@dPLGA. Subsequently, SIM@dPLGA was put into 20 mL SF protein solution (4 mg/mL) and mixed with a rotameter for 1 h. After adding 2 % glutaraldehyde (Acmec, China) for 0.5 h for cross-linking, the excess aldehyde groups were neutralized with glycine solution (Solarbio, China), washed sufficiently with pure water, and freeze-dried to obtain SF-SIM@dPLGA.

**Scanning electron microscope (SEM)**

The morphology of the particles was characterized by SEM (ZEISS, Germany) at an accelerating voltage of 2 kV. The groups of particles were placed uniformly on the conductive adhesive and the particles’ morphology was observed after gold spraying treatment. Energy Dispersive Spectroscopy (EDS) analysis was carried out during the period. 50 particles were randomly selected from the captured images and particle size was measured using Image J.

**Fourier transform infrared (FTIR) spectroscopy**

SIM, SF, dPLGA, SIM@dPLGA, SF-SIM@dPLGA were mixed with KBr under drying lamp. After grinding in a grinding bowl, FTIR spectra were completed under transmission mode using an FTIR spectrometer (Nicolet is-10, USA).

**X-ray diffraction (XRD) analysis**

An x-ray polycrystalline diffractometer instrument (Empyrean, Netherlands-Malvern-Panaco) was used for XRD examination, with a 2θ range of 5-90° for Cu Kα radiation and an XRD scan rate of 8°/min.

**Drug loading and encapsulation efficiency**

The drug loading and encapsulation efficiency of SIM in SIM@dPLGA and SF-SIM@dPLGA were determined by a UV spectrophotometer. 10 mg of both sets of particles were dissolved in 10 mL of dichloromethane. The diluted samples were detected by UV spectrophotometer at 238 nm. The drug loading and encapsulation efficiency were calculated according to the following equation:

Drug Loading = (weight of SIM in microparticles/weight of microparticles) × 100 %

Encapsulation efficiency = (Drug Loading/theoretical loading capacity) × 100 %

2.6. *In vitro* drug release

10 mg SIM@dPLGA or SF-SIM@dPLGA was dissolved in 2 mL 0.5% SDS/PBS release solution, mixed with ultrasonic waves and placed in a dialysis bag (8000-14000 D). The dialysis was then put into a 38 mL release solution. All samples were set into a thermostat at 100 rpm 37 °C. Assays were performed at predetermined time points, with 4 mL of release solution removed and replenished with fresh release solution each time. And then examined through a UV spectrophotometer. The drug release was calculated according to the following equation:

Drug Release = (Drug release/total drug content ) × 100 %

***In vitro* degradation**

10 mg of particles were accurately weighed and transferred to 10 mL PBS containing Proteinase k. All samples were placed in a constant temperature oscillator at 37 °C, and removed at a pre-determined time point, centrifuged, freeze-dried and weighed to calculate the degradation curve. During this process, the pH was examined with an acidimeter, and the pH curve was plotted as a function of time.

**Anti-erosion testing**

A simplified in vitro erosion model was developed to assess the capacity of particles to be retained. The procedure is as follows: 20 mg of each sample (PLGA microspheres, Bio-Oss^®^, SF-SIM@dPLGA) was evenly spread over an 8 mm cranial defect. The defect was then tilted at a 45° angle and 2 mL of PBS was poured from a constant height above the sample over a period of 10 seconds. Images of the sample were captured during the flushing process.

**Cytotoxicity test**

BMSCs were inoculated in 96-well plates, and then different particles were added to the plates after cell attachment. After 24, 48, or 72 h’s incubation, cell viability was detected and calculated through Cell Counting Kit-8 (Biosharp, China).

**Cell adhesion**

The adhesion of cells to the prepared particles was observed using confocal laser scanning microscopy (CLSM) (HITACHI, Japan). Briefly, cells incubated with different particles for 8 h and 24 h were fixed with 4 % paraformaldehyde, treated with 0.5 % Triton X-100, stained with phalloidin-FITC (Solarbio, China) for 30 min, covered with a blocking agent containing DAPI (Solarbio, China) and observed under CLSM.
 For quantitative analysis of cell adhesion, confocal laser scanning microscopy (CLSM) images were acquired from at least n = 3 independent samples per group. Using ImageJ software, the number of adherent cells per granule (or per unit area) was counted based on DAPI-stained nuclei. Data are presented as mean ± standard deviation (SD), and statistical comparisons were performed using two-way analysis of variance.

**Cell migration**

Cell migration was detected by cell scratch test. Photographs were taken at 0, 6, 12, and 24 h using a light microscope (Nikon, Japan). Cell migration rates were analyzed by Image J.

**Transwell migration assay**

The migratory response of rBMSCs to simvastatin (SIM) was evaluated using Transwell inserts (8 μm pore size, 24-well format). Briefly, rBMSCs were suspended in serum-free medium and seeded into the upper chambers at a density of 2×10^5^ cells per insert. The lower chambers were filled with complete medium containing SIM at different concentrations (0, 0.05, 0.1, 0.2, 0.5, and 1 μM). After incubation for 24 h at 37 °C with 5 % CO2, non-migrated cells on the upper surface of the membrane were gently removed using a cotton swab. Cells that migrated to the underside of the membrane were fixed with 4 % paraformaldehyde for 15 min and stained with 0.1 % crystal violet for 15 min. Membranes were washed with PBS, imaged under an inverted microscope, and migrated cells were quantified by counting cells in five randomly selected fields per insert.

**Osteogenic differentiation**

BMSCs were incubated with osteogenic induction medium for 7 days to complete ALP staining, and the staining procedure was performed according to an ALP kit (Beyotime, China). Cells were incubated with an osteogenic induction medium for 21 days to complete ARS staining, and the staining procedure was conducted using 0.2 % alizarin red S (Solarbio, China).

**qRT-PCR analysis**

BMSCs after different treatments were collected by 300 μL RNA lysate. Total RNA was extracted according to the instructions of an RNA extraction kit (Promega, China). The purity and concentration of RNA were determined using a Nanodrop 2000 spectrophotometer (Thermo, America). cDNA synthesis was conducted following the All-in-One First-Strand cDNA Synthesis SuperMix for qPCR (One-step gDNA Removal) kit instructions (TransGen Biotech, China). Amplification was conducted using the Green qRT-PCR SuperMix kit (Transgen Biotech, China) and signals were detected using a PCR system (Thermofisher, USA). The data were collated and analyzed using the 2^-ΔΔ^Ct method, with the internal reference gene *Gapdh* serving as a control. The primer sequences utilized in the qRT-PCR are presented in Table S2.

Table S2. Primers and sequences used in this experiment.

| Primer name | Sequence (5’ to 3’) |
| --- | --- |
| *Runx2*-F  *Runx2*-R  *Alp*-F  *Alp*-R  *Opn*-F  *Opn*-R  *Gapdh*-F  *Gapdh*-R | GACCAACCGAGTCATTTAAGGC  AAGAGGCTGTTTGACGCCAT  TGTCTCAAGATTGACAGGGCA  TCAATACCGGAAGGAGTGCTG  CTATGGCCGCTGGCAGTATC  CCAGCAATGACGTCCAGGAT  GCTCTCTGCTCCTCCCTGTT  CCGATACGGCCAAATCCGTT |

**Western blot analysis**

BMSCs after different treatments were collected in 300 μL protein lysate. The total protein concentration was determined by Nanodrop 2000, and then boiled, separated by SDS-PAGE, transferred onto polyvinylidene fluoride (PVDF) membranes, and blocked in skim milk. All membranes were incubated in the corresponding primary antibody overnight and in the secondary antibody for one hour, after which they were exposed to an imager (Tanon, China). Images were saved and analyzed by Image J.

**Micro-CT analysis**

The cranial bones of rats in each group were harvested after 4 or 8 weeks of observation, and each bone tissue was scanned using a micro-CT (Bruker, Germany). The data were reconstructed using CTAn software, and the related parameters, including BMD, BV/TV, trabecular number (Tb. N), and trabecular separation (Tb. Sp), were analyzed using CTvox and DataViewer.

**Histologic assays**

Cranial bones after micro-CT scanning were fixed with 4 % paraformaldehyde, decalcified using an EDTA decalcification solution and subsequently buried in paraffin wax. Sections of the samples were prepared for histological examination using Hematoxylin and eosin (H&E) staining and Masson trichrome staining. At the same time, important organs, such as the brain, liver, kidney, spleen, heart, and lung were also prepared for H&E staining.

**Coagulation assay**

The rat's tail was excised at a distance of 5 cm from the root of the tail after anesthesia, allowing the tail to naturally droop by gravity, and placed in air for 5 seconds to ensure normal blood loss. The rat's severed tail was then evenly dipped in different particles, and the coagulation time was recorded. The whole blood was collected in anticoagulation tubes and placed in a 37 °C water bath. Anticoagulated whole blood and different particles were combined before the addition of calcium ions. The EP tubes were rotated every 20 seconds to monitor for clotting and photographed for documentation.

**Statistical Analysis**

The statistical analyses were conducted using the t-test and one-way analysis of variance (ANOVA) in GraphPad Prism 10 software.
